# The effect of transcranial alternating current stimulation on cognitive flexibility and attention of children with intellectual disability: a case report

**DOI:** 10.1186/s13256-024-04625-w

**Published:** 2024-07-05

**Authors:** Zahra Ghahri Lalaklou, Elahe Haghighat-Manesh, AmirHossein Montazeri Ghahjavarestani, Ezzatollah Ahmadi

**Affiliations:** 1 Faculty of Psychology and Educational Sciences, Azarbaijan Shahid Madani University, Tabriz, Iran; 2 Department of Basic Sciences, School of Medicine, Zabol University of Medical Sciences, Zabol, Iran; 3 Universitat Autònoma de Barcelona, Barcelona, Spain

**Keywords:** Transcranial alternating current stimulation, Cognitive flexibility, Attention, Inhibition response, Children with intellectual disability, Case study

## Abstract

**Background:**

Intellectual disability is a neurodevelopmental disorder characterized by significant impairments in intellectual functioning and adaptive behavior. Cognitive flexibility and attention are crucial cognitive domains often affected in children with intellectual disability. This case report explores the novel use of transcranial alternating current stimulation, a noninvasive brain stimulation technique, to enhance these cognitive functions. The study’s novelty lies in its focus on alpha-wave frequency transcranial alternating current stimulation targeting specific Brodmann areas and its potential sustained impact on cognitive flexibility and attention in the pediatric population with intellectual disability.

**Case presentation:**

The case study involved two elementary school students, both 7 years old with mild intellectual disability, one male and one female, both with Turkic ethnicity, from Shahid Fahmideh School for Exceptional Children in Khosrowshah, Iran. Both participants underwent a 2-week intervention with daily 20-minute sessions of transcranial alternating current stimulation at an alpha-wave frequency (10 Hz), targeting Brodmann areas F3 and P3. Cognitive flexibility and attention were assessed using the Wisconsin Card Sorting Test and the Clock Test, administered at four time points: pre-intervention, week 1, week 2, and 1 month post-intervention. Statistical analysis showed significant improvements in both Wisconsin Card Sorting Test and Clock Test scores for both participants compared with baseline, with sustained enhancement over time.

**Conclusion:**

The findings from this case report indicate that transcranial alternating current stimulation may be a promising intervention for improving cognitive flexibility and attention in children with intellectual disability. The significant and sustained improvements observed suggest that transcranial alternating current stimulation could have a meaningful clinical impact on the cognitive development of this population. However, further research is needed to confirm the efficacy of transcranial alternating current stimulation and to explore its broader applicability and long-term effects in larger, more diverse populations.

## Background

Intellectual developmental disorder (IDD), also known as intellectual disability (ID), is a neurodevelopmental disorder characterized by significant impairments in intellectual functioning and adaptive behavior across multiple domains, including conceptual, social, and practical skills. These impairments typically manifest during the developmental period, typically before age 18 years. IDD is estimated to affect approximately 10 in 1000 individuals worldwide. Intellectual functioning is assessed using standardized tests of intelligence, with individuals with IDD typically scoring two or more standard deviations below the population mean. Academic difficulties are common in individuals with IDD, particularly in reading, writing, arithmetic, time management, and financial literacy. While IDD is a neurodevelopmental disorder, it should be distinguished from neurocognitive disorders, which involve a loss of cognitive functioning rather than a lifelong impairment [8]. Individuals with ID often exhibit impairments in executive functioning, a set of higher-order cognitive processes that govern planning, decision-making, and adaptability [3]. Cognitive flexibility, a crucial aspect of executive functioning, underpins creative thinking and manifests as the ability to adjust cognitive states in response to changing situations or task demands [1]. It encompasses the capacity to generate diverse perspectives on a specific problem or scenario and seamlessly transition between different modes of thought when confronted with stimuli that challenge existing cognitive patterns [16, 28]. Cognitive flexibility manifests in two primary forms: adaptive flexibility, signifying the ability to modify the mental framework through which solutions to problems are perceived, and automatic flexibility, representing the rapidity with which individuals produce various distinct ideas related to a given situation [15].

Various methods exist for evaluating cognitive flexibility, including employing neurocognitive assessments. The Wisconsin Card Sorting Test (WCST) stands as a prominent neurocognitive tool for assessing cognitive flexibility [20, 30]. This task demands that participants categorize a series of cards according to shifting rules and adapt their strategies accordingly when the rules unexpectedly change. Typically, individuals with reduced cognitive flexibility encounter difficulties in adjusting to evolving guidelines [12]. Navigating an increasingly complex world necessitates sophisticated cognitive control mechanisms. The abundance of information and potential actions demands efficient attention, decision-making, and coordination to avoid being overwhelmed. To manage this multiplicity and minimize confusion, evolution has equipped us with neural systems that integrate lower-level sensory and motor processes around a unifying theme, an internal goal. This capacity for cognitive control undoubtedly involves extensive neural circuitry, but the prefrontal cortex (PFC) is widely recognized as a key player [21]. Attention, another constituent of executive functions, encompasses all aspects of human cognition that an individual can regulate, as well as all cognitive processes associated with limited resources or capacity management. Attention can be further categorized into selective attention, sustained attention, and self-regulatory attention [27]. The selective attention of participants was assessed using the Clock Test, requiring them to attend to the ticking of a clock and respond whenever a beat was skipped. The data on reaction time during the task served to gauge the individual’s attentional levels [23]; a cross-platform, open-source programming language was employed to create the experimental protocol. As cognitive flexibility and attention are critical for intellectual functioning, transcranial alternating current stimulation (tACS) was implemented to enhance these domains in children with intellectual disability.

Transcranial alternating current stimulation is a noninvasive brain stimulation (NIBS) technique that has gained widespread popularity over the past decade. This method involves the direct application of alternating electric currents to the scalp, allowing it to modulate neural activity primarily in the cerebral cortex [9]. tACS typically employs a sinusoidal waveform, characterized by a gradual transition between positive and negative voltage values over each half-cycle. Consequently, the current direction alternates between anodal and cathodal electrodes, corresponding to the two halves of the waveform [9, 14]. The underlying concept of tACS is to mimic the brain’s naturally occurring rhythmic patterns of electrophysiological activity, as observed through electroencephalography (EEG) and magnetoencephalography (MEG). The cost, portability, tolerability, and safety profiles of tACS are superior to other noninvasive stimulation techniques. This makes tACS a feasible tool for modulating intrinsic brain rhythms and associated brain functions without imposing additional excitatory or inhibitory burdens [9].

The mechanism of tACS is characterized by four main modes of action: first, frequency entrainment, which occurs when the tACS frequency matches that of the endogenous oscillations, resulting in the stimulation wave coinciding with the endogenous wave at a consistent phase across cycles [5]; second, rhythm resonance, where the stimulation frequency directly influences the endogenous oscillation, potentially facilitating synchronization; third, temporal biasing of spikes, where the interaction between the stimulation and internal currents modulates the timing of neural firing, potentially enhancing the activation of specific neuronal populations during each stimulation cycle; fourth, network entrainment, where strong tACS amplitudes can entrain irregular neural activity, and imposed pattern, where exceptionally strong stimulation is required to override endogenous oscillations and induce a new oscillatory pattern [9, 19]. tACS has emerged as a promising brain stimulation technique for elucidating the neural mechanisms underlying various cognitive domains, including conflict and error processing, and enhancing cognitive performance, such as working memory and long-term memory [2, 11]. tACS is a noninvasive brain stimulation method that allows to directly modulate brain oscillations of a given frequency. Using this method, it was recently shown that increasing alpha (10 Hz) oscillations improved creative ideation with figural material [10]. Considering the extensive body of research demonstrating the efficacy of tACS in cognitive and psychiatric domains, this study investigates the potential of tACS to modulate cognitive flexibility and attention in children with intellectual disability.

## Case presentation

The case study focuses on two elementary-school students diagnosed with mild intellectual disability (ID), referred to as patient 1 (male) and patient 2 (female), both 7 year old, with Turkic ethnicity, attending Shahid Fahmideh School for Exceptional Children in Khosrowshah, Iran. The study aimed to investigate the potential of transcranial alternating current stimulation (tACS) in enhancing cognitive flexibility and attention in children with ID. Table 1 describes patient information.

**Table 1:** Descriptive patient information

Patient	Demographic details	Medical history and symptoms	Treatment/intervention	Outcomes
Patient 1 (male)	7-Year-old elementary school student with mild intellectual disability	Diagnosed with mild intellectual disability, exhibiting challenges in cognitive flexibility and attention	Underwent a 2-week intervention involving daily 20-minute sessions of tACS at an alpha-wave frequency (10 Hz), targeting Brodmann areas F3 and P3	WCST and Clock Test scores showed improved cognitive flexibility and attention; intervention reduced errors and response times across all points, suggesting lasting benefits
Patient 2 (female)				

The diagnosis was confirmed through a general assessment before entering the primary education level (age 6–6.5 years) followed by a specialized intelligence test administered by the Education Organization. Both participants met the diagnostic criteria for ID and were enrolled in special schools for children with ID. Notably, they did not receive any formal intervention for ID prior to enrollment in the special education program.

Family interviews identified a history of delayed verbal development in both children. The boy’s first words emerged after 3 years old, while the girl’s emerged at 2 years 8 months.

The first child’s medical history revealed a maternal age of 28 years and a paternal age of 42 years at conception. Delivery occurred via cesarean section at 37 weeks gestation. This child is the second-born, with a 13-year-old brother diagnosed with intellectual disability, hyperactivity, and attention deficit. A history of intellectual disability is present in the maternal lineage (maternal grandmother); however, the limited access to specialized diagnosis and education precludes the determination of its severity.

The second child’s mother was 41 years old, and the father was 49 at conception. Delivery occurred naturally at 39 weeks gestation. This child is the third-born, with older siblings aged 23 and 17 years who have no history of intellectual disability. No familial history of genetic disorders was reported.

Physical examination revealed no abnormal craniofacial features or skin pigmentation in either child. Specialist evaluations for Down syndrome, Fragile X syndrome, and Prader–Willi syndrome were not conducted. Owing to the segregation of educational settings for intellectual disability and autism spectrum disorder (ASD), both children underwent ASD screening, which yielded negative results.

A neurological examination revealed myopia diagnosed at age 7 in patient 1 (who presently wears glasses). No hearing, vision, or movement impairments were detected in patient 2.

## Materials and methods

The type of research method is a case study, a quasi-experimental research design in which a subject’s change in the dependent variable is measured [7]. Participants were drawn from a population of second-grade children with mild intellectual disability attending Shahid Fahmideh School for Exceptional Children in Khosrowshah, Iran, during the 2019–2020 academic year (*n* = 9). Two students (one male, one female) meeting inclusion criteria (parental consent, IQ of 50–70, normal sensory/movement function, adequate comprehension, and successful baseline tasks) and lacking exclusion criteria (non-cooperation from family or participants, sensory problems such as deafness and blindness and movement problems such as finger problems) were selected. tACS targeting Brodmann areas F3 (left medial prefrontal cortex) and P3 (left lateral parietal cortex) [31] was employed at 10 Hz, coinciding with the alpha frequency band. This alpha-band stimulation (10 Hz) was administered for 20 minutes daily, six days per week, across two consecutive weeks (12 sessions in total). This specific protocol aimed to influence cognitive flexibility and attention in children with intellectual disability. The tools and tests that are utilized are introduced in the following.

### Wisconsin Card Sorting Test

**Fig. 1:**
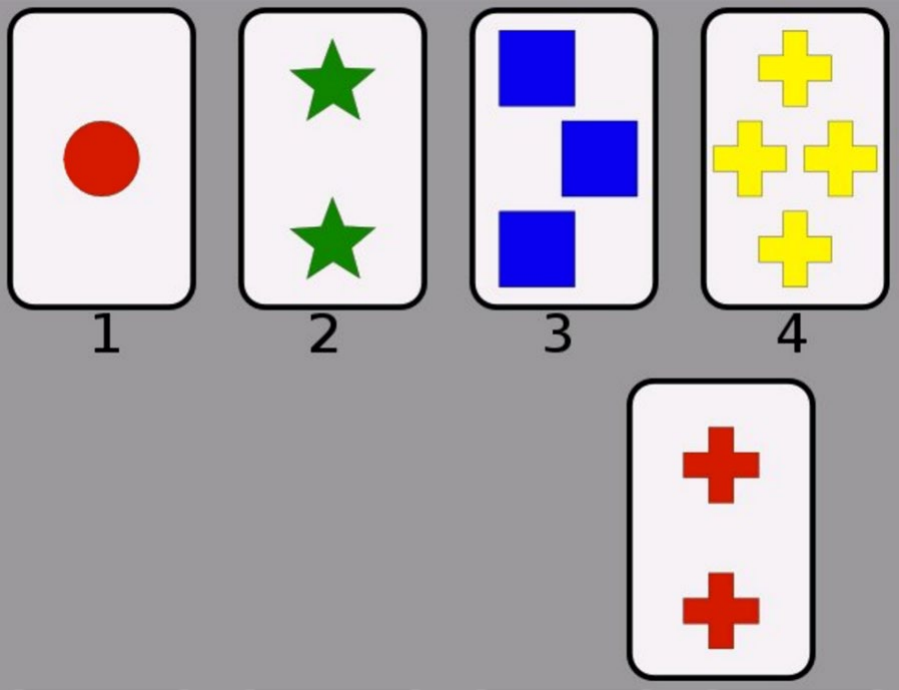
The Wisconsin Card Sorting Test

Cognitive flexibility was assessed using the computerized WCST, a 128-card version, obtained from the psychology experiment building language (PEBL) platform (Fig. 1). In this task, participants sorted 128 cards with one of the four key cards according to one of three predefined criteria (color, shape, or number). The criterion changed periodically without prior notice, requiring participants to adapt their sorting strategy on the basis of feedback. The WCST demonstrates strong validity (> 0.86) for measuring cognitive deficits after brain damage [17], and its reliability in the Iranian population has been reported at 0.85 using the retest method [22].

### Mackworth Clock Test

**Fig. 2:**
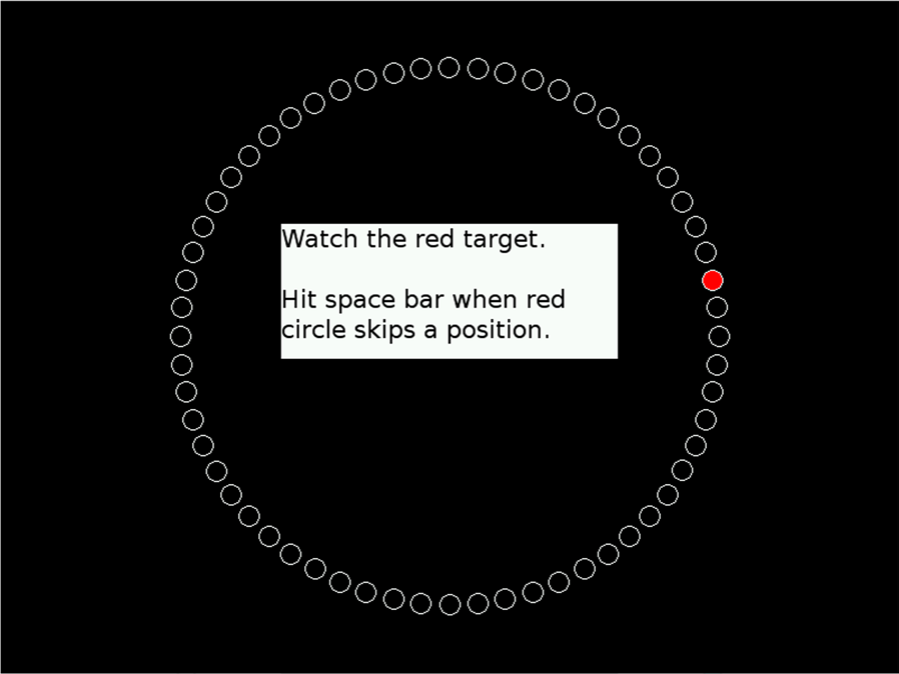
Mackworth Clock Test

The Clock Test (Mackworth, 1948) assesses sustained visual attention. Participants monitor a large, circular clock-like display with a black-and-white pointer making short jumps every second, like the second hand of an analog clock (Fig. 2). The light jumps to another circle at irregular intervals, occurring 12 times in 30 s. The task requires pressing a button upon detecting double jumps. The subject watches the clock and determines when it passes a beat. The hand advances in discrete steps per second, completing a group of 100 steps of the circle. Participants, typically seated 50 cm from the screen, press a key upon detecting a double jump. Responses within 8 seconds are scored as hits, missed signals within 8 seconds as misses, and premature responses as false alarms. The test offers customizable parameters (test length, signal jump distance) via a preferences window, allowing for flexible experimental designs. Durations of 30 minutes, 1 hour, or 2-minute practice sessions are available, and signal jump distance can be doubled or quadrupled. Upon completion, the test automatically displays a completion message and provides a detailed results summary through a dedicated window [18]. This test, instrumental in understanding continuous attention and alertness, exhibits a significant decline in signal detection accuracy after 30 minutes of continuous performance. Various forms, including computerized versions, remain relevant in research, particularly for assessing sobriety.

### Methodology

The study employed a mixed-methods approach to evaluate the effectiveness of the implemented interventions. Descriptive statistics (frequency, percentage, mean, standard deviation, min/max) were used to characterize the research findings. To assess the statistical stability of observed pre–post intervention changes, a visual analysis method and the reliable change index (RCI) were implemented. The RCI, calculated using Eq. 1, determines whether observed changes exceed measurement error, thereby indicating actual versus spurious effects. In this formula, the difference between the post-test score of each subject and their pre-test score is divided by twice the square of the measurement standard error [13].

1 $$\text{RCI}=\frac{{\text{X}}_{\text{post}}-{\text{X}}_{\text{pre}}}{\sqrt{2{\text{SE}}^{2}}}.$$

The standard deviation [29] error was also calculated using Eq. 2:

2 $$\text{SE}=\text{SD}\sqrt{1-\text{r}}$$

The present study employed a two-pronged approach to assess the efficacy of transcranial electrical brain stimulation with alternating current on the cognitive flexibility and attention of children with intellectual disability. Firstly, a statistical threshold based on the *z*-score (1.96) was used to determine the significance of pre-test to post-test score changes. This ensured that observed improvements exceeded chance findings with 95% confidence. Secondly, the recovery percentage formula (Eq. 3) adapted from Ref. [7] was applied to quantify the intervention’s impact. A recovery percentage exceeding 30% for pre-test versus post-test scores (both immediate and follow-up) served as an indicator of a positive and durable effect.

3 $$\text{RP}=\frac{{\text{X}}_{\text{post}}-{\text{X}}_{\text{pre}}}{{\text{X}}_{\text{pre}}}\times 100.$$

This combined approach strengthens the reliability and generalizability of the findings, offering valuable insights into the potential of tACS as a therapeutic tool for this population.

### Intervention protocol

Prior to the intervention, baseline data were collected for two participants (one male, one female). This included quantitative electroencephalography (QEEG) brain mapping, computerized versions of the WCST and Clock Test, and demographic information. Subsequently, participants received tACS at 10 Hz (alpha frequency) for 20 minutes daily, six days per week, for two consecutive weeks (12 sessions in total).

Phase 1 (sessions 1–6):

Bilateral tACS targeted the F3 and P3 brain regions using a Neurostim device with elastic head and arm straps, carbon electrodes, and disposable sponge pads. Alpha wave stimulation (10 Hz) was administered for 20 minutes per session. WCST and Clock Tests were administered after the sixth session.

Phase 2 (sessions 7–12):

tACS administration continued under the same protocol (frequency, duration, and target regions) as in phase 1. WCST and Clock Tests were performed after the 12th session, determining the changes in brain waves, and the data were recorded.

Post-intervention:

One month after the final tACS session, participants underwent follow-up assessments to evaluate the intervention’s long-term impact and stability. These included executive function tests and QEEG brain mapping.

## Results

This single case study involved two participants, a 7-year-old boy and a 7-year-old girl, diagnosed with mild intellectual disability at 4 and 3 years old, respectively. Their continuous attention was assessed using the Clock Test (Table 2).

**Table 2:** Descriptive index, the mean scores of the continuous attention variable in the Clock Test in the subjects

Subject	Indicator	Pre-test	Post-test		Follow-up
			End of the first week	End of the second week	
First subject (male)	Reaction time (ms)	470	475	451	461
	Correct answer	26	29	31	28
	Wrong answer	34	31	29	32
Second subject (female)	Reaction time (ms)	590	572.6	583.25	570.75
	Correct answer	32	42	46	45
	Wrong answer	28	18	14	15

Table 2 summarizes the observed changes in reaction time and accuracy for both subjects. Pre-test scores serve as baseline measurements. Results indicate that both participants exhibited improvements in attention after the intervention. The boy showed increasing average correct answers (26 to 31) and decreasing average incorrect answers (34 to 29) on post-tests and follow-up. His average reaction time also improved across assessments. Similarly, the girl displayed higher average correct answers (32 to 46) and lower average incorrect answers (28 to 14) post-intervention. Her reaction time decreased consistently from the pre-test to the follow-up.

Table 3 presents the stability index of the attention variable for further analysis.

**Table 3:** Indicators of stability in the subjects’ attention variable in the Clock Test

Subject	Indicator	Pre-test to post-test first week	Pre-test to post-test second week	Pre-test to follow-up	Post-test first week to post-test second week	Post-test second week to follow-up
First subject (male)	Reaction time (ms)	0.2	−0.79	−0.37	−1	0.41
	Correct answer	2.5	4.16	1.66	1.66	−2.5
	Wrong answer	−2.5	−4.16	−1.66	−1.66	2.5
Second subject (female)	Reaction time (ms)	−0.73	−0.28	−0.8	0.44	−0.52
	Correct answer	8.33	11.66	10.83	3.33	−0.83
	Wrong answer	−8.33	−11.66	−10.83	−3.33	0.83

Table 3 presents the stability index for continuous attention (Clock Test) across pre-test, post-test, and follow-up assessments for both subjects. Both subjects displayed improvements in attention stability as indicated by positive changes in correct answers and reduced negative changes in reaction time and incorrect answers across assessments. The boy’s stability index for correct answers reached statistical significance for all comparisons versus the pre-test. Similarly, the girl’s stability index for correct answers exceeded the threshold for significance (> 1.96) in post-test week 2 and follow-up. These findings suggest sustained improvements in attention following the intervention.

In Table 4, the results of the attention recovery percentage are given.

**Table 4:** Percentage of improvement of attention in the Clock Test

Subject	Indicator	Post-test (first week)	Post-test (second week)	Follow-up
First subject (male)	Reaction time (ms)	−1.06	4.04	1.91
	Correct answer	11.53	19.23	7.69
	Wrong answer	8.82	14.70	5.88
Second subject (female)	Reaction time (ms)	2.94	1.14	3.26
	Correct answer	31.25	43.75	40.62
	Wrong answer	35.71	50	46.42

Table 4 demonstrates the percentage improvement in attention for both subjects following tACS intervention. Compared with baseline (pre-test), significant gains were observed at week 1 and week 2 in several parameters. These findings suggest a positive effect of tACS on sustained attention performance.

In Table 5, the descriptive data of the cognitive flexibility variable of the subjects are given.

**Table 5:** Descriptive index and mean cognitive flexibility variable in the Wisconsin Card Sorting Test

Subject	Indicator	Pre-test	Post-test (first week)	Post-test (second week)	Follow-up
First subject (male)	Correct responses	41	35	77	70
	Total errors	87	93	51	58
	Perseverative errors	86	0	36	42
	Nonperseverative errors	1	93	15	16
	Unique errors	0	0	0	0
	Conceptual level responses	10	3	59	38
Second subject (female)	Correct responses	34	60	76	79
	Total errors	94	68	52	49
	Perseverative errors	0	64	17	0
	Nonperseverative errors	94	4	35	49
	Unique errors	4	2	0	1
	Conceptual level responses	11	42	56	53

Table 5 reveals significant improvements in cognitive flexibility for both subjects. Compared with baseline (pre-test), the boy demonstrated sustained enhancement in both correct answers (increased post-test and follow-up) and conceptual level responses (similarly increased). The girl likewise exhibited significant gains in correct answers across all post-test and follow-up assessments, and her conceptual level scores also improved in these assessments. These findings suggest a positive impact of tACS on cognitive flexibility in both subjects.

Table 6 presents the stability index of the subjects’ cognitive flexibility variable.

**Table 6:** Stability indicators of the subjects’ cognitive flexibility in the Wisconsin Card Sorting Test

Subject	Indicator	Pre-test to post-test first week	Pre-test to post-test second week	Pre-test to follow-up	Post-test first week to post-test second week	Post-test second week to follow-up
First subject (male)	Correct responses	−1.68	10.08	8.12	11.76	−1.96
	Total errors	1.68	−10.08	−8.12	−11.76	1.96
	Nonperseverative errors	1.94	0.15	0.31	−1.64	0.02
	Unique errors	0	0	0	0	0
	Conceptual level responses	−13.72	96.09	54.91	109.82	−41.18
Second subject (female)	Correct responses	7.28	11.76	12.60	4.48	0.84
	Total errors	−7.28	−11.76	−12.60	−4.48	−0.84
	Nonperseverative errors	−1.90	−1.24	−0.94	0.65	0.29
	Unique errors	−0.98	−1.96	−1.47	−0.98	0.49
	Conceptual level responses	60.79	88.25	82.36	27.45	−5.88

Table 6 reveals significant improvement in subject 1’s conceptual level scores on the Wisconsin Test, persisting through the follow-up stage. Additionally, the absolute value of the stability index for subject 1’s correct answers and total errors exceeded 1.96 in all comparisons between the pre-test and subsequent assessments (post-test, follow-up). This indicates statistically significant and stable changes in these scores following the intervention. Similarly, subject 2 demonstrated significant gains in conceptual level scores throughout the study period, with stable changes in both correct answers and total errors across pre-test, post-test, and follow-up comparisons (absolute stability index > 1.96). Notably, subject 2 also exhibited a stable change in unique errors between the pre-test and second-week post-test. These findings support the hypothesis that tACS has a positive impact on cognitive flexibility, as evidenced by enhanced performance at both the conceptual level and other aspects of the Wisconsin Test. The randomized nature of the attention test and Wisconsin cards, coupled with the time interval between tests and the participants’ age, suggests a low likelihood of a training effect influencing the outcomes. Table 7 presents the results of the cognitive flexibility recovery percentage.

**Table 7:** The recovery percentage of cognitive flexibility in the Wisconsin Card Sorting Test

Subject	Indicator	Post-test (first week)	Post-test (second week)	Follow-up
First subject (male)	Correct answers	−14.63	87.80	70.73
	Total errors	−6.89	41.37	33.33
	Nonperseverative errors	−9.2	−1.4	−1.5
	Unique errors	0	0	0
	Conceptual level responses	70	−49	−28
Second subject (female)	Correct answers	0	0	−35.13
	Total errors	−76.47	12.52	47.87
	Nonperseverative errors	0	44.68	47.87
	Unique errors	27.65	62.76	75
	Conceptual level responses	95.74	100	−38.81

Table 7 highlights notable gains in cognitive flexibility for both subjects. Compared with baseline (pre-test), recovery percentages exceeded 30% across all subsequent assessments (post-test weeks 1 and 2, follow-up). This finding suggests a potentially potent impact of tACS on cognitive flexibility in both participants.

## Discussion

This study aims to examine the effect of tACS on the cognitive flexibility and attention of children with intellectual disability. In the first hypothesis, the results showed that tACS had a significant effect on the attention of children with intellectual disabilities in such a way that the amount of attention when comparing the pre-test score with the post-test of the first and second week and follow-up was more than 30%. This is in the direction of improvement, which shows the positive effect of tACS on the subjects’ attention. No studies have been published to investigate the effectiveness of tACS on the cognitive flexibility and attention of ID children as in the current study. Still, this result is consistent with one study that applied a single session of 40 Hz tACS over F3/F4, with an improvement in inhibitory control, visual attention, processing speed, and working memory [2]. Additionally, a case study employing a home-based tACS protocol involving 70 40-Hz stimulation over the left angular gyrus revealed significant improvements in memory, executive function, and attention. Notably, these benefits were maintained over a 3-month follow-up period. This extended protocol also provided valuable insights into treatment tolerability and patient compliance, demonstrating 100% adherence without adverse events or severe side effects. In a separate case report, a 15-session 40-Hz stimulation regimen targeting the dorsolateral prefrontal cortex (DLPFC) and contralateral supraorbital area (SOA) led to improvements in memory, visuospatial abilities, executive function, attention, orientation, and verbal recall. These enhancements persisted even after a 4-month follow-up [2]. A previous study demonstrated a significant effect of transcranial direct current stimulation (tDCS) on sustained attention, with the active tDCS group exhibiting improved performance over repeated sessions compared with the sham group. Measurable active–sham differences emerged by the third treatment visit, suggesting a synergistic effect between CT and tDCS with repeated administration. Similar findings of improved attention following 12–16 hours of CT training have been reported in individuals with fetal alcohol spectrum disorders [4]. A comprehensive meta-analysis encompassing 102 published tACS studies involving 2893 participants yielded compelling evidence of cognitive enhancement across nine distinct cognitive domains. Notably, substantial improvements were observed in executive control, with moderate to large effect sizes. Additionally, mild to modest gains were detected in attention, long-term memory, working memory, and fluid intelligence. Taken together, these findings underscore the potential utility of tACS as both an investigational tool for deciphering the neurophysiological underpinnings of cognition and a rehabilitative intervention for cognitive enhancement [6].

Consistent with the second hypothesis, our results demonstrated a significant positive effect of tACS on cognitive flexibility in children with intellectual disability. This finding corroborates previous research investigating the impact of tDCS on decision-making and cognitive flexibility in individuals with gambling disorder, where tDCS applied bilaterally over the dorsolateral prefrontal cortex (DLPFC; F3/F4) was shown to enhance both decision-making and cognitive flexibility in this population [26]. A systematic review of existing research findings indicates that tACS can effectively ameliorate symptoms of attention deficit hyperactivity disorder (ADHD), particularly cognitive impairments [24]. Notably, a clinical trial demonstrated the positive impact of an eight-session tDCS regimen combined with cognitive training on working memory, verbal fluency, and immediate and delayed recall [25].

Multicentre double-blind clinical trials with larger sample sizes are warranted to further investigate the efficacy of tACS in children with intellectual disability. These trials could also address the potential utility of different stimulation protocols, either across individuals or within the same participants, to evaluate the specificity of any observed effects and to explore potential synergies between different stimulation parameters. Further well-designed double-blind controlled trials with larger sample sizes and extended follow-up periods are essential to replicate the current findings and to identify reliable response predictors while controlling for various confounding factors. In this context, integrating electrophysiological recordings and modeling techniques is encouraged to optimize stimulation protocols and elucidate the underlying mechanisms mediating the effects of tACS.

## Conclusion

This single case study investigated the potential of tACS in improving executive functions and brain waves in two children with intellectual disabilities (both aged 7 years) in Khosrowshah, Iran (2019–2020). Participants received daily 20-minute alpha-wave (10 Hz) tACS, targeting Brodmann areas F3 and P3, sessions for two consecutive weeks, with pre- and post-intervention assessments. Quantitative electroencephalography brain maps and cognitive performance measures (WCST, Clock Test) were obtained at four time points: pre-intervention, post-week 1, post-week 2, and 1 month follow-up. Stability indexes and recovery percentages were calculated to analyze the response to the intervention.

Results revealed significant and stable improvements in both subjects. Notably, incorrect responses on the WCST and Clock Test decreased across all comparisons (pre- to post-week 1, pre- to post-week 2, post-week 2 to follow-up), exceeding the stability index threshold (> 1.96) for both mean error and response time. These findings suggest a stable improvement in cognitive performance, particularly in cognitive flexibility and sustained attention.

Limitations include the small sample size (two purposefully selected children), limiting generalizability, and potential moderating factors like socioeconomic status and family dynamics (uncontrolled variables). Additionally, the 2-week intervention duration might need to be increased for sustained effects.

Future research with more extensive, diverse samples and extended intervention periods is recommended to solidify these preliminary findings. Additionally, controlling for moderating variables and comparing participants based on socioeconomic and family characteristics could enhance result consistency.

This study contributes to the growing body of literature exploring noninvasive brain stimulation as a potential tool for enhancing cognitive function in children with intellectual disabilities. While further research is warranted, these initial findings hold promise for developing novel therapeutic interventions in this population.

## Data Availability

All data generated or analyzed during this study are included in this published article.

## Abbreviations

ADHD: Attention deficit hyperactivity disorder
DLPFC: Dorsolateral prefrontal cortex
EEG: Electroencephalography
ID: Intellectual disability
IDD: Intellectual developmental disorder
MEG: Magnetoencephalography
NIBS: Noninvasive brain stimulation
PFC: Prefrontal cortex
QEEG: Quantitative electroencephalography
RCI: Reliable change index
SOA: Supraorbital area
tACS: Transcranial alternating current stimulation
tDCS: Transcranial direct current stimulation
WCST: Wisconsin Card Sorting Test
